# Nanoparticle-Encapsulated Plant Polyphenols and Flavonoids as an Enhanced Delivery System for Anti-Acne Therapy

**DOI:** 10.3390/ph18020209

**Published:** 2025-02-04

**Authors:** Ririn Puspadewi, Tiana Milanda, Muhaimin Muhaimin, Anis Yohana Chaerunisaa

**Affiliations:** 1 Doctoral Program of Pharmacy, Faculty of Pharmacy, Padjadjaran University, Sumedang 45363, Indonesia; ririn22003@mail.unpad.ac.id; 2 Faculty of Pharmacy, Jenderal Achmad Yani University, Cimahi 40531, Indonesia; 3 Department of Pharmaceutical Biology, Faculty of Pharmacy, Padjadjaran University, Sumedang 45363, Indonesia; 4 Center of Herbal Studies, Padjadjaran University, Sumedang 45363, Indonesia; 5 Department of Pharmaceutics and Pharmaceutical Technology, Faculty of Pharmacy, Padjadjaran University, Sumedang 45363, Indonesia

**Keywords:** polyphenols, flavonoids, nanoparticles, anti-acne, medication delivery, skin penetration, nanotechnology, inflammation, antimicrobial properties

## Abstract

This study conducted a literature review by searching for articles related to the treatment of skin infections/wrinkles using nano-delivery systems containing natural compounds. The search was conducted in various databases for articles published in the last 10 years, with strict inclusion and exclusion criteria. Of the 490 articles found, 40 were considered relevant. *Acne vulgaris* is a common dermatological disorder characterised by inflammation of the sebaceous glands, often resulting in the development of pimples, cysts, and scarring. Conventional treatments, including antibiotics and topical retinoids, frequently demonstrate limitations such as side effects, resistance, and insufficient skin absorption. Recent advancements in nanotechnology have enabled the creation of innovative drug-delivery systems that enhance the effectiveness and reduce the adverse effects of anti-acne medications. Polyphenols and flavonoids, natural bioactive compounds with notable anti-inflammatory, antioxidant, and antibacterial properties, are recognised for their therapeutic effectiveness in acne treatment. However, their practical application is hindered by insufficient solubility, stability, and bioavailability. The incorporation of these compounds into nanoparticle-based delivery systems has shown promise in resolving these challenges. Various nanoparticle platforms, including lipid-based nanoparticles, polymeric nanoparticles, and solid lipid nanoparticles, are evaluated for their ability to improve the stability, controlled release, and targeted delivery of polyphenols and flavonoids to the skin. The advent of polyphenol and flavonoid-loaded nanoparticles marks a new acne therapy era.

## 1. Introduction

*Acne vulgaris* is a prevalent dermatological condition impacting teenagers and young adults, with an occurrence rate of 80–90% in these demographics [1]. This ailment is marked by the presence of comedones, papules, pustules, and nodules, frequently resulting in considerable psychological effects, including diminished self-esteem and anxiety problems [2]. While rarely life-threatening, the social and emotional repercussions of *Acne vulgaris* can profoundly impact the quality of life of individuals affected. Consequently, the advancement of efficacious and safe treatments for this condition has gained paramount significance in the domains of medicine and dermatology [3].

Conventional therapies for *Acne vulgaris* often include topical and systemic antibiotics, retinoids, and desiccants that diminish sebum production. Although numerous people observe enhancements, these interventions are not universally efficacious for every individual [4]. Furthermore, prolonged antibiotic treatment might result in bacterial resistance, whilst retinoids may have adverse effects such as skin irritation and increased susceptibility to sunlight. These problems underscore the pressing necessity for safer and more efficacious alternative therapies that may more comprehensively meet patients’ requirements (Leung et al., 2020) [5].

Recently, there has been an increasing interest in utilising natural chemicals such polyphenols and flavonoids as treatment agents for *Acne vulgaris*. Polyphenols, present in numerous plant sources, are recognised for their antioxidant, anti-inflammatory, and antibacterial characteristics [6]. Flavonoids, a subclass of polyphenols, exhibit potential efficacy in mitigating the aetiology of acne by diminishing inflammation and inhibiting the proliferation of *Propionibacterium acnes* [7]. The use of these chemicals provides a more natural and potentially safer method for managing *Acne vulgaris*, instilling new hope for patients in search of more effective therapies [8].

Notwithstanding the considerable promise of polyphenols and flavonoids in acne therapy, a difficulty exists due to the poor absorption of these substances when used topically or systemically [6]. Therefore, novel tactics are necessary to improve the efficacy of these medicines. An intriguing option that has developed is the utilisation of nanoparticles as medication delivery systems. Nanoparticles enhance the stability, bioavailability, and skin penetration of polyphenols and flavonoids, rendering them a compelling choice for the creation of more efficacious medicinal formulations [9,10].

Moreover, nanoparticle delivery technologies not only improve the efficiency of polyphenol and flavonoid administration but also have the capacity to alter the physicochemical features of these compounds, potentially augmenting their therapeutic usefulness [11,12]. Researchers can optimise the interactions between nanoparticles and the skin by altering their size, shape, and charge, thus minimising undesirable side effects. This discovery fosters optimism for more effective and safer acne treatments while fulfilling the demand for more integrated and eco-friendly therapeutic approaches [13,14].

This article seeks to investigate the efficacy of nanoparticle-encapsulated polyphenols and flavonoids as an advanced delivery mechanism for the treatment of *Acne vulgaris*. We will examine the advantages, obstacles, and recent advancements in this delivery method through a review of existing research, along with its therapeutic implications for enhancing the quality of life for patients with *Acne vulgaris*.

## 2. Method

The technique for the literature review entailed searching for papers in databases including PubMed, Scopus, Google Scholar, ScienceDirect, and Web of Science. Only articles published during the past decade were included to ensure relevance and current information. The search criteria employed were: (“skin infection” AND “bacterial infection” OR “*Staphylococcus aureus*” OR “*Propionibacterium acnes*”) AND (“natural compounds” OR “essential oils” OR “flavonoids”) AND (“nano carriers” OR “nanoparticles” OR “nano delivery systems”) AND (“topical cream” OR “non-nano formulation” OR “antibacterial cream” OR “conventional antibacterial treatments”) AND (“wound healing” OR “reduction of bacterial load” OR “skin recovery” OR “skin safety” OR “treatment efficacy”) AND (“clinical trials” OR “preclinical studies” OR “in vivo” OR “in vitro”) AND (“nano carrier” OR “nano formulation” OR “skin infection treatment”). The inclusion criteria specified articles that reported clinical or preclinical studies concerning nano delivery systems for treating skin infections utilising natural compounds. Exclusion criteria: review papers lacking original data, studies unrelated to skin infection treatment, and publications in languages other than English. Of the 490 articles retrieved, 40 were deemed relevant following the screening of titles and abstracts. A total of ten pertinent and detailed articles will be selected for this examination. Article selection necessitates a thorough consideration of technique, results, and conclusions to ensure the inclusion of only high-quality studies in the review.

## 3. Pathological Acne

**Figure 1:**
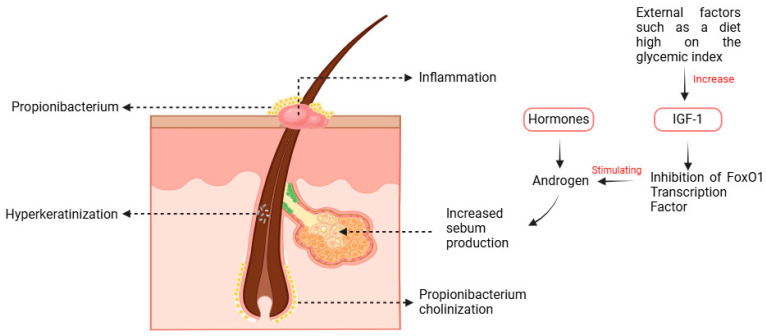
Demonstrates the pathogenesis of acne, encompassing multiple contributing components. Hyperkeratinization leads to follicular obstruction, while heightened sebum production and the proliferation of *Propionibacterium* intensify inflammation. Androgen hormones elevate sebum production, while growth factor IGF-1, affected by a high glycaemic index diet, promotes these hormones. The suppression of the FoxO1 transcription factor contributes to the exacerbation of acne by elevating inflammation and sebum production. All these elements together induce the production of acne (Created with Biorender.com).

The pathophysiology of acne is multifaceted, involving complicated mechanisms related to changes in sebum production, aberrant keratinisation, and an immunological response due to bacterial infection (Figure 1). These result in symptoms such as comedones, papules, pustules, or nodulocystic lesions, significantly affecting the physical appearance and the quality of life of those affected [15,16].

A crucial aspect in the development of acne is that androgens are a class of hormones that promote the growth and function of sebaceous glands. Elevated androgen levels may result in the proliferation of sebocytes and heightened sebum production [17,18]. Excessive sebum, rich in highly saturated fatty acids, may clog hair follicles, potentially leading to comedone production. Moreover, insulin-like growth factor 1 modulates elevated lipid production and heightened androgen receptor activity, hence exacerbating the inflammatory potential in acne [19,20].

Another key element in the pathophysiology of acne is excessive keratinisation. This induces keratinocytes to proliferate excessively, resulting in the formation of plugs that obstruct the follicle. Factors contributing to this may include an elevation in interleukin-1α levels generated by *Propionibacterium acnes*, which enhances the likelihood of hyperkeratinization. The buildup of keratin in the follicle not only hinders sebum flow but also fosters an environment conducive to bacterial growth, especially *Propionibacterium acnes*, which acts as an opportunistic pathogen in acne formation [17,18,21,22].

The interaction with the immune system is a crucial element of *Propionibacterium acnes*. It can provoke inflammatory responses by activating TLRs on keratinocytes and sebocytes, leading these cells to release the pro-inflammatory cytokines IL-6 and IL-8, which exacerbate inflammation and cause tissue damage at the lesion site. Evidence also indicates dysbiosis among *Propionibacterium acnes*. The inflammation of acne may be intensified; certain subtypes may play a more significant role in the aetiology of acne than others [23,24,25].

Another significant aspect is environmental influences and food habits. High glycaemic diets and milk consumption have been linked to increased acne severity, potentially due to IGF-1 activation. Conversely, omega-3 fatty acids and low glycaemic diets demonstrate preventive effects against acne formation. Consequently, lifestyle modifications and dietary alterations can aid in the management and prevention of the condition [26,27,28].

## 4. Conventional Products as Anti-Acne Agents

The primary objective of acne treatment is to manage and address existing acne lesions, avoid permanent scarring to the greatest extent feasible, reduce the length of the condition, and minimise morbidity. The patient should be apprised of the objectives related to preventing new acne lesions while facilitating the healing of existing ones. Patients should be informed that progress may take 3 to 6 weeks to become evident [29]. Some conventional products that have been used as anti-acne can be seen in the following table, Table 1.

## 5. Polyphenols and Flavonoids as Anti-Acne Agents

Presently, acne therapies frequently utilise synthetic compounds that, although efficacious, may result in adverse side effects. As a result, researchers are exploring natural remedies that provide enhanced safety and efficacy, with polyphenols and flavonoids identified as leading contenders [10,26,35].

**Figure 2:**
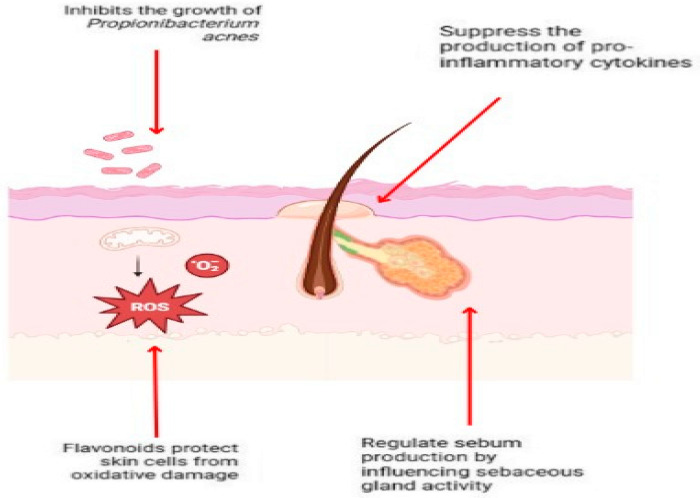
Demonstrates the method by which flavonoids attack acne. Flavonoids confer multiple preventive benefits to the skin; primarily, they suppress the proliferation of *Propionibacterium acnes*, a bacterium implicated in the pathogenesis of acne. Secondly, flavonoids inhibit the synthesis of pro-inflammatory cytokines, hence mitigating inflammation in the skin. Third, flavonoids safeguard skin cells from oxidative injury by diminishing the production of Reactive Oxygen Species (ROS). Fourth, flavonoids modulate sebum production by affecting sebaceous gland function, hence aiding in the prevention of pore obstruction and acne development. These systems collaboratively function to alleviate symptoms and avert acne (Created with Biorender.com).

Polyphenols and flavonoids are bioactive substances present in many plant sources, such as fruits, vegetables, and tea. They are renowned for their various health advantages, encompassing antioxidant, anti-inflammatory, and antibacterial attributes. Recent research indicate that these two categories of chemicals can significantly contribute to the treatment of acne. Utilising the inherent properties of polyphenols and flavonoids can mitigate acne symptoms and avert future occurrences [7,36]. Figure 2 shows demonstrates the method by which flavonoids attack acne.

The methods by which polyphenols and flavonoids mitigate acne encompass multiple interrelated biological pathways, including:

1. Mitigation of Inflammation: Polyphenols and flavonoids can inhibit the synthesis of pro-inflammatory cytokines, which are integral to the inflammatory response. This alleviates the erythema and oedema linked to acne lesions [37].
2. Antimicrobial Activity: These substances demonstrate antimicrobial activities, decreasing the proliferation of *Propionibacterium acnes*, the bacteria implicated in acne formation [38].
3. Regulation of Sebum Production: Polyphenols and flavonoids can modulate sebum production by affecting sebaceous gland function, consequently diminishing pore obstruction [8].
4. Free Radical Scavenging: Polyphenols and flavonoids function as scavengers of free radicals, safeguarding skin cells from oxidative damage that may aggravate skin diseases [39,40].

Polyphenols and flavonoids help regulate sebum, which is a crucial element in acne development. These bioactive chemicals can alter sebum (skin oil) production by acting on many pathways that govern the activity of the sebaceous glands [6]. Polyphenols and flavonoids can diminish excessive sebum production by regulating the function of sebaceous glands. Excessive sebum is a primary contributor to acne development since it can obstruct pores, creating an optimal habitat for bacteria such as *Propionibacterium acnes* to proliferate [41]. Additionally, polyphenols and flavonoids influence inflammatory pathways by lowering oxidative stress and pro-inflammatory cytokines, which both lead to hyperactive sebaceous glands. They can aid in preventing excessive sebum production, which frequently leads to acne flare-ups, by lowering inflammation [39].

One important factor that leads to acne is sebum oxidation. Oxidised sebum can block pores, cause inflammation, and encourage the growth of microorganisms. Because of their antioxidant qualities, polyphenols and flavonoids can help counteract free radicals created when sebum oxidises, halting the inflammatory chain reaction that causes acne. Polyphenols and flavonoids can shield skin cells from oxidative damage in addition to shielding sebum from oxidation, which helps to avoid irritation and clogging that can worsen acne [42].

The benefits of polyphenols and flavonoids as anti-acne agents stem not only from their efficacy but also from their safety profile. The utilisation of these natural chemicals offers numerous advantages over synthetic treatments, which frequently correlate with detrimental side effects [43].

A key advantage of polyphenols and flavonoids is their superior safety profile. In contrast to numerous synthetic drugs that may induce irritation or allergic responses, polyphenols and flavonoids are typically regarded as milder and more compatible with the skin. This renders them a more secure option, particularly for persons with delicate skin who may be unable to endure traditional treatments [44].

An additional benefit is their sustained effectiveness. The application of polyphenols and flavonoids not only alleviates existing acne symptoms but also functions as a prophylactic strategy to diminish future occurrences. These chemicals can assist sustain skin microbiota equilibrium and avert acne recurrence by diminishing inflammation and enhancing overall skin health [45].

The synergistic potential among diverse polyphenols and flavonoids can augment therapy efficacy. The amalgamation of many bioactive substances inside a singular formulation can yield more potent and expansive benefits, facilitating a comprehensive approach to acne therapy. Utilising green tea extracts abundant in epigallocatechin gallate (EGCG) alongside other flavonoids can enhance anti-inflammatory and antibacterial properties, offering improved skin protection [46,47].

In a progressively health-conscious and sustainable age, the utilisation of plant-based products rich in polyphenols and flavonoids satisfies the demand for natural remedies while promoting sustainable agriculture methods. This corresponds with the global trend of diminishing dependence on synthetic chemicals and transitioning towards more eco-friendly alternatives [48].

In light of these factors, it is evident that polyphenols and flavonoids provide a novel and sustainable method for acne treatment. Through continued research and the development of successful formulations, it is anticipated that these natural substances will gain greater recognition and application in acne treatments in the future. Table 2 below informs mechanisms of flavonoid and polyphenol compounds in the antibacterial and anti-inflammatory treatment of acne.

Polyphenols and flavonoids are bioactive compounds found in a variety of plants, which are known for their antioxidant and anti-inflammatory properties. Although it promises to be an anti-acne agent, there are some limitations in its efficacy:

1. Bioavailability: The absorption and metabolism of polyphenols and flavonoids can be restricted. Many of these compounds have low bioavailability, which means only a small amount reaches the systemic circulation or target tissue, thus reducing their potential effectiveness [56].
2. Stability: Polyphenols and flavonoids can be sensitive to light, heat, and oxygen. This instability can lead to a decrease in its efficacy when exposed to environmental factors, making it less reliable in topical formulations [57].
3. Concentration: Effective anti-acne activity often requires a high concentration of active compounds. Achieving these concentrations in formulations may not be practical or safe for skin applications [58].
4. Mechanism of Action: The exact mechanism by which polyphenols and flavonoids exert anti-acne effects is not fully understood. More research is needed to clarify how they interact with skin cells and acne-causing bacteria [6].
5. Individual Variability: The effectiveness of these compounds can vary among individuals due to genetic differences, skin type, and the presence of other skin conditions, which can affect how the skin responds to treatment [59].
6. Potential Allergic Reactions: Some people may experience allergic reactions or skin irritation due to topical use of products rich in polyphenols or flavonoids, thus limiting their use [60].
7. Limited Clinical Evidence: Although some studies have shown benefits, there is a need for more rigorous clinical trials to confirm the effectiveness of polyphenols and flavonoids as anti-acne agents and to establish optimal dosage and formulation [61].

## 6. Nanoparticles as Delivery Systems for Polyphenols and Flavonoids

The growing interest in natural compounds for medicinal use has focused much emphasis on polyphenols and flavonoids, recognised for their various health benefits, such as antioxidant, anti-inflammatory, and antibacterial capabilities. The therapeutic use of these bioactive chemicals is frequently hindered by their limited solubility, insufficient stability, and inadequate bioavailability. Innovative delivery strategies are necessary to improve therapeutic efficacy in response to these obstacles. Nanoparticles have emerged as effective carriers for polyphenols and flavonoids, providing numerous benefits that enhance their efficacy in diverse applications, especially in dermatology [9,62,63].

Nanoparticles are characterised as entities with diameters between 1 and 100 nm. They can be fabricated from diverse materials, including lipids, polymers, metals, and ceramics, each imparting distinct characteristics that can be customised for certain delivery purposes. Lipid-based nanoparticles, including solid lipid nanoparticles (SLNs) and nanostructured lipid carriers (NLCs), are preferred due to their biocompatibility and capacity to encapsulate hydrophobic substances. Polymeric nanoparticles, derived from natural or synthetic polymers, have the benefit of customisable surface properties that can affect drug release kinetics and cellular uptake [64,65].

Metallic nanoparticles, especially silver and gold, have garnered interest due to their inherent qualities, such as antibacterial efficacy and visual attributes, which can augment the therapeutic effects of encapsulated drugs. Moreover, biodegradable nanoparticles offer an eco-friendly alternative, guaranteeing low toxicity and safe decomposition inside biological systems. The diversity of nanoparticle forms facilitates a customised strategy for optimising the distribution of polyphenols and flavonoids, hence augmenting their therapeutic efficacy [66,67,68].

Enhancing the skin penetration and stability of polyphenols and flavonoids is essential for improving their efficacy. The stratum corneum, the outermost layer of the epidermis, serves as a robust barrier that restricts the infiltration of substances. Nanoparticles can enhance transdermal delivery in various ways. Their diminutive size enables them to infiltrate the intercellular lipid regions of the stratum corneum. Furthermore, nanoparticles can be designed with surface changes, including functionalisation with penetration enhancers, which can compromise the lipid barrier and facilitate enhanced permeability [69,70,71].

The variability in sources of natural bioactive compounds, including polyphenols and flavonoids, can be mitigated through various strategies to enhance the consistency of nanoparticle formulations [72]. Standardising the extraction procedures and source materials is crucial to lowering variability. To maintain a constant concentration and composition of the bioactive substances, this may entail the use of particular plant species, harvest schedules, and regulated growing conditions [73]. Isolating and purifying the active compounds before formulation guarantees a consistent starting material. Characterisation techniques such as High-Performance Liquid Chromatography (HPLC) and Mass Spectrometry (MS) are utilised to assess the purity and consistency of bioactive compounds [74].

The stability and release profile of the bioactive compounds can be made more predictable by modifying the formulation parameters, such as the surfactant levels, the composition of the nanoparticle matrix, and the preparation technique (e.g., solvent evaporation or emulsification), which will lessen batch-to-batch variations [75].

**Figure 3:**
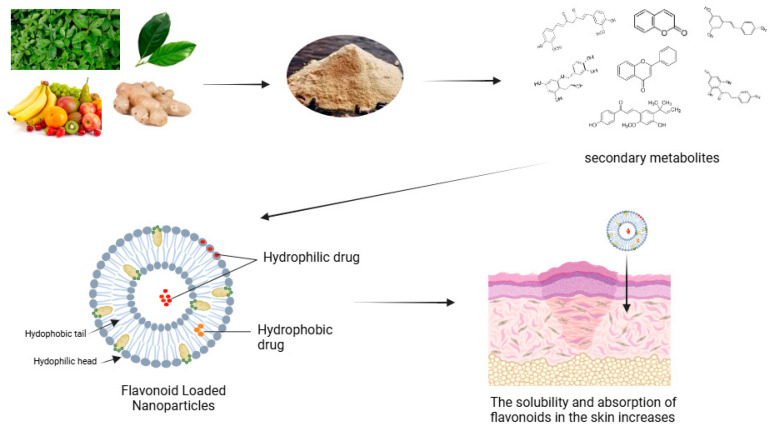
Formulation process of flavonoid nanoparticles to augment absorption and efficacy of dermatological treatments (Created with Biorender.com).

Nanoparticles promote skin permeation by utilising physical and chemical methods to modify skin permeability. Certain nanoparticles can function as micro-needles, generating temporary micro-channels in the skin, thereby facilitating the improved distribution of encapsulated substances. Moreover, the use of nanoparticles alongside physical techniques like ultrasound or iontophoresis can significantly augment penetration, offering a non-invasive strategy for drug delivery [13,76]. The diagram demonstrates how this system encapsulates both hydrophilic and hydrophobic medicines, facilitating effective transdermal distribution as depicted in Figure 3.

Stability is a crucial element affecting the efficacy of polyphenols and flavonoids. These chemicals are susceptible to deterioration from environmental conditions like light, heat, and oxygen, potentially resulting in reduced therapeutic efficacy. Nanoparticle encapsulation safeguards these chemicals from degradation, hence improving their stability during storage and application. This protection is especially critical in topical formulations, since exposure to air and light may undermine product integrity [13].

Although there are several advantages to utilising nanoparticles for the delivery of polyphenols and flavonoids, potential side effects and toxicity issues must be considered. The physicochemical characteristics of nanoparticles, such as size, shape, surface charge, and composition, are essential in assessing their biocompatibility. Although numerous studies indicate that nanoparticles may be well tolerated, extensive toxicological evaluations are still necessary to assess the long-term effects of exposure to these delivery methods [77].

Alongside evaluating cytotoxicity, researchers must account for the possibility of systemic absorption of nanoparticles. The inadvertent introduction of nanoparticles into the bloodstream may result in unforeseen repercussions, including detrimental effects on organs and systems. Consequently, it is imperative to engineer nanoparticles that improve delivery while concurrently reducing toxicity risks through meticulous design and thorough testing [78]. Figure 3 shows formulation process of flavonoid nanoparticles to augment absorption and efficacy of dermatological treatments.

Nanoparticle platforms have varying characteristics, which affect their ability to improve the stability and controlled release of polyphenols and flavonoids for topical administration applications [79]. Lipid nanocarriers have wide applications in topical drug delivery due to their biocompatible, biodegradable, non-toxic, and non-irritating lipid properties [80].

The following is a specific summary of the most commonly used lipid-based nanosystems, and their applications as nanocarriers for the encapsulation of phenolic compounds in dermal/transdermal delivery.

### 6.1. Liposome

In terms of structure, liposomes are spherical particles that encircle the interior of an aqueous compartment with one or more phospholipid layers [81,82]. The vesicular structure, arising from the amphipathic characteristics of the bilayer-forming lipids, enables the encapsulation of both hydrophobic and hydrophilic molecules [83]. The physicochemical qualities of liposomes (size, surface charge, etc.), membrane properties, and the interaction between the encapsulated active agent and liposomal components affect the method and efficacy of drug administration [83,84]. The optimal size of liposomes for topical application is less than 300 nm to penetrate deeper skin layers. Vesicles smaller than 70 nm exhibit optimal deposition in both the epidermis and the dermis [85]. Liposomes transfer active agents to the skin through various mechanisms, including vesicle fusion with stratum corneum lipids, fluidizing effect, intact penetration into dermal layers, and improved drug delivery through hair follicles or sweat ducts [86,87,88]. Phan et al. investigated the interaction mechanism between two distinct kinds of polyphenols, namely flavonoids and trans-stilbenes, and liposomal membranes. The gallate, galloyl, and hydroxyl groups of flavonoids are linked to membrane lipids through hydrogen bonding, resulting in a compact phospholipid structure, less surface area, and the formation of a more rigid bilayer. The benzyl open-ring configuration of trans-stilbenes facilitates their profound intercalation into the hydrophobic bilayer, resulting in an expansion of the membrane area and an increase in fluidity [89].

### 6.2. Nanostuctured Lipid Carriers

Nanostructured lipid carriers consist of solid and liquid lipids distributed in an aqueous phase, stabilised by surfactants [90]. The incorporation of a liquid lipid into their composition destroys the highly organised crystalline structure typical of the SLNs. It results in a disordered lipid matrix, facilitating increased drug accumulation [91,92]. Loo et al. investigated the effects of different lipid concentrations, the ratio of lipid to oil, and the addition of propylene glycol and lecithin on skin hydration, transepidermal water loss, and the long-term physical stability of nanostructured lipid nanocarriers. The conclusion indicates that NLCs with elevated lipid content, solid lipid content, phospholipids, and lecithin constitute a highly effective delivery mechanism for topical cosmetic applications aimed at enhancing skin hydration [93].

### 6.3. Solid Lipid Nanoparticles

Solid lipid nanoparticles (SLNs) consist of singular or a combination of lipids that remain solid at both ambient and physiological temperatures, distributed in water or an aqueous phase containing surfactants [94]. The lipids most frequently employed in the formulation of solid lipid nanoparticles encompass triglycerides such as trimyristin and tristearin, fatty acids including stearic and palmitic acid, various waxes like beeswax and cetyl palmitate [95]. Based on their structural characteristics and drug localisation, these can be categorised into homogenous matrix models and core–shell models, which include drug-enriched shells and drug-enriched cores [96]. The characteristics of solid lipid nanoparticles including their composition and physicochemical properties, influence their permeation through the skin. The lipid characteristics of SLNs suggest several mechanisms for skin penetration, similar to those of liposomes. These include the fusion of nanoparticles with the lipids of the stratum corneum, the lipid fluidizing effects of lipids, and transfollicular transfer. Nonetheless, the specific penetration mechanism associated with SLNs may suggest their occlusive effect. Their extensive surface area and nanoscale dimensions confer occlusive properties, resulting in improved skin hydration and enhanced penetration into dermal layers [97]. Kakkar et al. conducted a study utilising tetrahydrocurcumin-loaded solid lipid nanoparticles (SLNs) that demonstrated adequate occlusivity and anti-inflammatory properties. Their additional integration into hydrogel formulation resulted in a skin permeation ability that was seventeen times greater than that of plain tetrahydrocurcumin gel [98].

### 6.4. Nanoemulsions

Nanoemulsions are colloidal dispersions that are isotropic and consist of water and oil. They are stabilised with a surfactant or cosurfactant. One of the liquids is dispersed into nanosized particles, which are typically between 20 and 200 nm in size [99]. Nanoemulsions represent a promising drug-delivery system for topical applications, characterised by their small droplet size, uniform size distribution, and extensive surface area. These properties contribute to their effective spreading on the skin surface, thereby enhancing drug penetration [100]. Su et al. investigated the transport of nanoemulsions containing environment-responsive and fluorescent dyes through the skin. Their findings indicate that formulations with droplet sizes of 80 nm can diffuse into the uncompromised epidermis and traverse the canals of hair follicles, unlike larger formulations with droplet sizes of 500 nm [101]. The encapsulation of hydrophilic molecules in w/o nanoemulsions may enhance transdermal transport due to the solubilising effects of surfactants on the stratum corneum, facilitating delivery through pore pathways and hair follicle canals for larger molecules [102].

It is important to focus on the safety and the long-lasting compatibility of nanoparticle systems when using them to give helpful compounds like polyphenols and flavonoids in skincare and treatment products [62]. Several strategies can be employed, including the following:

- Use nanoparticles made from biocompatible and biodegradable materials, such as polymers or lipids, to minimise long-term accumulation in the body [103].
- Alter the nanoparticle surface to augment biocompatibility, diminish toxicity, and enhance targeting efficacy. This may entail the application of biocompatible polymers or ligands that enhance cellular absorption and diminish immune response [104].
- Perform thorough in vitro investigations to evaluate cytotoxicity, cellular uptake, and immune responses prior to in vivo application [105].
- Conduct comprehensive animal studies to assess the long-term effects, distribution, metabolism, and excretion of nanoparticles [105].
- Long-term monitoring of nanoparticle systems in clinical application should focus on adverse effects and efficacy [106].

## 7. Current Studies on Polyphenol and Flavonoid-Loaded Nanoparticles in Acne Treatment

Nanoparticle formulation is a crucial research domain focused on enhancing the delivery of polyphenols and flavonoids for acne therapy. Diverse procedures have been established for the fabrication of nanoparticles that contain bioactive chemicals, including solvent evaporation, coacervation, and electrospinning processes. Each approach presents unique benefits and obstacles, affecting the dimensions, stability, and release characteristics of the resultant nanoparticles. Solvent evaporation techniques are esteemed for their capacity to generate nanoparticles with regulated dimensions and morphology, whereas electrospinning facilitates the creation of nanofibers that can function as drug-delivery systems [107].

The selection of the formulation technique profoundly influences the effectiveness of nanoparticles in transporting active chemicals to the intended location. Research indicates that particle size, surface charge, and material composition are critical determinants of nanoparticles’ interactions with skin cells and their subsequent penetration through the skin barrier. Nanoparticles of a smaller size, often between 100 and 200 nm, demonstrate superior penetration capacities, crucial for attaining therapeutic concentrations in the dermis where acne lesions develop [108].

Numerous studies have emphasised the necessity of evaluating both in vitro performance and in vivo outcomes when comparing the efficacy and delivery metrics of polyphenol and flavonoid-loaded nanoparticles. Efficacy measurements typically encompass the diminution of inflammation, microbial burden, and total acne lesions. Delivery metrics concentrate on the pace and extent of drug release, the depth of skin penetration, and the duration of activity. These thorough assessments allow researchers to derive significant findings about the most successful formulations [109].

Recent clinical and preclinical investigations have shown significant data, reinforcing the therapeutic potential of nanoparticles loaded with polyphenols and flavonoids in the treatment of acne. Randomised controlled research indicated that a quercetin-loaded nanoparticle formulation significantly reduced acne lesions compared to conventional topical therapies. Furthermore, preclinical investigations employing polyphenol-rich extracts encapsulated in lipid-based nanoparticles showed increased antibacterial efficacy against *Propionibacterium acnes*, the principal pathogen associated with acne formation [110].

Furthermore, the safety profile of nanoparticle formulations is critically significant, particularly for their prolonged application in the management of chronic disorders such as acne. Research on the cytotoxicity and skin irritation potential of polyphenol and flavonoid-loaded nanoparticles has yielded encouraging findings, demonstrating that these compositions possess low toxicity and negligible adverse effects, rendering them appropriate for daily use. This feature is especially attractive for patients desiring effective treatments devoid of the undesirable responses linked to conventional therapy [111].

The development of nanoparticles encapsulating polyphenols and flavonoids enhances therapeutic effectiveness while conforming to the increasing preference for sustainable and natural treatment alternatives. Patients are increasingly favouring formulations sourced from natural origins, and the integration of sustainable practises in nanoparticle manufacturing can improve consumer acceptance and marketability. Investigation into biodegradable polymers and natural surfactants for nanoparticle formulation is accelerating, aiding the advancement of eco-friendly skincare products [112].

Continuous study is crucial to clarify the mechanisms of action that contribute to the improved effectiveness of these nanoparticles as the field progresses. Comprehending the interactions of polyphenols and flavonoids at the cellular and molecular levels in relation to acne pathogenesis will yield significant insights for the formulation of more tailored therapeutics. This fundamental understanding will facilitate the optimisation of formulation strategies and the enhancement of delivery systems to maximise treatment effects [113].

Numerous bioactive chemicals, such as flavonoids and polyphenols, have been formulated into nanoparticle forms to augment their effectiveness in therapies, including acne therapy. Table 2 delineates an array of flavonoids and polyphenols synthesised as nanoparticles, including the interventions implemented, the categories of nanocarriers employed, and the findings derived from the research.

### 7.1. Curcumin

Curcumin, the active ingredient in turmeric, has been produced as nanoparticles (nanocurcumin) via the wet-milling technique. The wet-milling approach involves dissolving 100 mg of curcumin in dichloromethane, spraying it into boiling water under ultrasonic settings, and subsequently concentrating and freeze-drying to produce nanocurcumin powder. Nanocarriers based on polymers are utilised for the delivery of nanocurcumin. The study revealed that nanocurcumin had superior antibacterial activity compared to its conventional form, as indicated by minimum inhibitory concentrations against various bacterial and fungal species [114].

### 7.2. Resveratrol

Resveratrol, a flavonoid with antioxidant properties, was included into nanoemulsion gel formulations comprising adapalene and resveratrol. Two formulations were administered: the first with 5 mg of resveratrol (F1) and the second with 10 mg of resveratrol (F2), both taken once daily. These nanoemulsions were formulated utilising a surfactant-based nanocarrier to improve the solubility and penetration of the active chemical. The formulated nanoemulsion gel containing adapalene and resveratrol showed superior performance in treating acne pustules compared to the commercial formulation, with significantly reduced treatment duration [115].

### 7.3. Quercetin

Quercetin, a flavonoid recognised for its antioxidant properties, has been formulated in various nanostructures to enhance its absorption and efficacy. The used nanocarriers comprise lipid-based variants, including liposomes and nanostructured lipid carriers (NLCs), which enhance the oral absorption, targeting, and antioxidant efficacy of quercetin. Lipid-based nanocarriers significantly improve the bioavailability and efficacy of quercetin, rendering it a viable choice for acne treatment and other dermatological disorders [110].

### 7.4. Mangosteen

Mangosteen, possessing anti-inflammatory and antibacterial capabilities, was formulated as a 0.5% mangosteen nanoparticle-loaded gel (MNLG). This gel was applied to one side of the face twice a day, in conjunction with a 1% clindamycin gel. Lipid and polymer-based nanocarriers were utilised in this formulation of a gel containing mangosteen. The findings indicated that MNLG markedly diminished comedone and inflammatory acne lesions by 66.86% and 67.05%, respectively, following 12 weeks of treatment, equal to the 55.33% and 64.16% reduction observed with 1% clindamycin gel. MNLG demonstrated a markedly superior enhancement in clinical severity vs. to the 1% clindamycin gel at week 12 [116].

Mangosteen, namely mangostin, was utilised as a mangostin nanoparticle gel at a concentration of 1.2% mangostin. Application occurred bi-daily following showering for a duration of 28 days. Lipid-based nanocarriers were employed in this gel composition. The mangostin nanoparticle gel exhibited a markedly greater enhancement in both the acne severity index (ASI) and inflammatory lesion count compared to the placebo gel, hence suggesting its efficacy in acne treatment [113]. Table 3 informs efficacy of flavonoid and polyphenol nanoparticle formulations in acne treatment.

## 8. Mechanisms of Improved Anti-Acne Effectiveness via Nanoparticles

The key mechanism contributing to the efficacy of nanoparticles in acne treatment is enhanced skin penetration and bioavailability. Nanoparticles are defined by their minuscule dimensions, allowing for more effective penetration of the epidermal layers than traditional formulations. This ideal size diminishes skin penetration barriers, enabling active substances to access target cells more swiftly and efficiently. Studies indicate that lipid and polymeric nanoparticles can improve the topical absorption of active drugs, resulting in a decreased onset time and heightened drug concentration at targeted areas [62].

Improved bioavailability is essential for therapeutic efficacy and can help mitigate systemic adverse effects. By administering medications directly to the targeted region, nanoparticles reduce the quantity of medication required to attain optimal clinical results. This not only reduces treatment expenses but also enhances patient comfort, a crucial element in therapy adherence. Consequently, nanoparticles not only augment efficacy but also enhance the safety profile of acne treatments [119].

Moreover, nanoparticles provide prolonged and regulated release patterns, which are critical components of acne therapy. Numerous conventional formulations necessitate frequent applications owing to fast and erratic drug release. Nonetheless, nanoparticles can be engineered to release pharmaceuticals progressively, sustaining therapeutic amounts for prolonged durations. This facilitates more uniform therapy and diminishes application frequency, which is especially advantageous for patients with hectic daily schedules [120]

By means of meticulous design and alteration, nanoparticles can regulate the rate and pattern of medication release, thereby augmenting the overall efficacy of treatment. For example, nanoparticles can be altered with diverse components, including pH or temperature-responsive polymers, to facilitate a more precise medication release in inflammatory regions deeper within the skin. This method not only improves drug absorption but also customises treatment responses to individual patient requirements [120].

Moreover, nanoparticles can synergise with conventional acne therapies, enhancing the total therapeutic efficacy. The integration of nanoparticles with traditional drugs like retinoids or antibiotics can yield supplementary advantages, improving therapy efficacy by concurrently targeting many pathogenic pathways of acne. This combination has the potential to enhance clinical results and diminish the risk of resistance commonly associated with monotherapy [121].

The incorporation of nanoparticles into therapy protocols can improve tolerability. A multitude of patients experience considerable adverse effects from conventional acne therapies, including dermal irritation and erythema. The utilisation of nanoparticles allows for fewer dosages to attain equivalent outcomes, hence decreasing the probability of adverse responses. This is essential for enhancing patients’ quality of life, frequently undermined by the adverse effects of therapies [122].

Conversely, additional study is required to comprehensively elucidate the mechanisms underlying the effectiveness of nanoparticles in acne treatment. Despite multiple studies demonstrating favourable results, heterogeneity in nanoparticle formulations and properties can affect outcomes. Consequently, standardised methodologies and stringent evaluation processes must be implemented to guarantee the safety and efficacy of nanoparticle-based goods in the marketplace [122].

The integration of these pathways underscores the considerable potential of nanoparticles to revolutionise acne treatment. Ongoing research and the advancement of novel formulations may render nanoparticles an essential element in the treatment arsenal against acne. Consequently, it is imperative to conduct additional research on the interactions between nanoparticles and skin tissues, together with the physical properties influencing penetration and drug release, to optimise the efficacy of this therapy [123].

The development of multi-functional nanoparticles that possess a combination of properties, such as anti-inflammatory, antimicrobial, and wound-healing capabilities, could substantially enhance the treatment outcomes for *Acne vulgaris* in numerous ways [124]. Nanoparticles are sufficiently small to permeate the skin and concentrate on the sebaceous glands and hair follicles that are responsible for the development of acne. It is possible to directly deliver each therapeutic property to the site of inflammation, infection, or injury by integrating multiple functions into a single nanoparticle. This could result in a more efficient and effective treatment with fewer systemic adverse effects [111].

*Acne vulgaris* is usually linked to inflammation from blocked pores and the body’s immune reaction. By including anti-inflammatory agents like corticosteroids in nanoparticles, you can manage the release of these agents directly where they are needed. This helps reduce heat, swelling, and pain in the skin while minimising the risk of too much absorption into the body [122]. Acne can cause scars and slow down the repair of skin spots. Adding mending substances like growth factors or special peptides to nanoparticles can help skin heal more quickly and reduce scarring. Patients with inflammatory acne, whose lesions may take longer to heal or leave scars after acne, might benefit most from this.

Conventional therapies, such as oral antibiotics or topical retinoids, may produce adverse consequences, including antibiotic resistance or dermal irritation. Utilising nanoparticles for localised and controlled release of active substances minimises systemic exposure, hence diminishing potential negative effects while enhancing efficacy at the treatment site [111].

## 9. Challenges and Future Perspectives

In the age of swift medical advancement, the emergence of nanoformulation has become a symbol of optimism in the therapeutic domain. This technology provides a more efficient method for drug delivery, especially for intricate diseases like cancer and neurological disorders. Nanoformulation, with its distinctive capacity to augment bioavailability and optimise drug release characteristics, offers viable remedies for some challenges encountered by traditional therapies. Nonetheless, despite considerable advancements, numerous hurdles must be resolved prior to the widespread adoption of this technology in clinical practice [125].

A principal problem in the development of nanoformulations is the intricacy of the formulation process. The synthesis of nanoparticles necessitates a comprehensive comprehension of the interactions among active compounds, nanomaterial matrices, and the adjacent biological milieu. Constraints in synthesis and characterisation techniques may result in inconsistencies in the quality of the final product, thereby impacting the treatment’s efficacy and safety. This variability presents a difficulty for developers and offers a risk to patients dependent on these therapies [126].

A notable problem is reproducibility, defined as the capacity to reliably generate the same product across various batches. In a meticulously standardised environment, uniformity is paramount. If nanoparticles cannot be consistently generated, their clinical applications will be impeded. This issue is especially significant considering that nanotechnology is very nascent, with numerous synthesis processes still under development [127].

In light of these limitations, there is fresh optimism regarding the possibilities for personalised treatment. Personalised medicine has emerged as a prominent trend, wherein medications are customised to meet individual requirements based on genetic traits, environmental influences, and lifestyle decisions. Utilising nanotechnology, we can develop therapies that are more targeted and precise. This method not only improves therapeutic efficacy but also diminishes adverse side effects. Specifically engineered nanoparticles can more effectively target cancer cells, preserving healthy tissues and reducing the negative effects commonly linked to traditional medicines [128].

The integration of nanotechnology with a tailored methodology offers a significant opportunity to develop safer and more efficacious treatment protocols. Nevertheless, considerable and inventive research is needed to do this. Customised nanoparticle-based strategies that can adjust to the individual requirements of patients will be a primary emphasis for future advancement. Comprehensive study in this domain could provide significant insights into optimising treatments and enhancing treatment outcomes for patients [129].

The advancement of nanoparticle-encapsulated plant polyphenols and flavonoids as an improved delivery mechanism for anti-acne treatment presents considerable potential, although numerous hurdles persist. A key challenge is maintaining the stability and bioavailability of these natural substances when encapsulated in nanoparticles. Plant polyphenols and flavonoids frequently exhibit inadequate solubility and breakdown under specific conditions, which may impede their efficacy in therapeutic applications [130].

The creation of nanoparticles that are biocompatible and proficient in encapsulating these chemicals continues to be a challenging endeavour. Optimising particle size, surface charge, and drug release kinetics is essential for enhancing treatment efficacy and reducing side effects. Moreover, the risk of skin irritation or allergic responses to nanoparticle compositions requires meticulous assessment, particularly for sensitive skin related to acne [131].

In the future, the advancement of nanotechnology methods, including intelligent nanoparticles that release active substances in reaction to environmental stimuli (e.g., pH, temperature), may enhance the accuracy of medication administration. Additional investigation into the synergistic interactions of polyphenols and flavonoids alongside nanoparticles is crucial for developing more efficacious anti-acne therapies, providing tailored treatment options, and meeting the increasing demand for natural, non-invasive alternatives in dermatology [132].

The following compounds are a group of polyphenols/phenols and flavonoids that have the potential to be developed in the treatment of acne.

### 9.1. Coumarin

Prior research has demonstrated that solid lipid nanoparticles may significantly contribute to the encapsulation of coumarin derivatives for the treatment of various bacterial infections, including those caused by methicillin-resistant *Staphylococcus aureus*. This research indicates that coumarin-encapsulated solid lipid nanoparticles can successfully address MRSA infections [133]. Although the findings presented are encouraging with respect to therapies for bacterial infections, investigations concerning the use of coumarin nanoparticles as a treatment for acne remain notably scarce. Despite being recognised for its anti-inflammatory and antimicrobial properties, which could be very useful in the treatment of acne, nano-coumarin has not been extensively studied in acne treatment. This paved the way for further studies directed toward formulating coumarin nanoparticles as a more effective anti-acne treatment by improving the penetration and stability of the active ingredient in the skin.

### 9.2. Lichocalcone

Lichocalcone, extracted from the Liquorice plant, exhibits anti-inflammatory and antibacterial properties, positioning it as a viable option for acne treatment [52]. Despite the absence of studies on lichocalcone nanoparticles, its bioactive qualities indicate potential as a promising drug if formulated in nanoparticle form. Investigating the application of lichocalcone nanoparticles for acne therapy may provide novel opportunities for effective cosmetics formulations.

### 9.3. Hesperidin

Hesperidin has been documented to exhibit considerable antimicrobial, anti-inflammatory, and antioxidant activities [43]. The development of nanoformulations of hesperidin has been extensively investigated due to its potential therapeutic effects, with the goal of improving its bioavailability and targeted administration. Prior research has predominantly concentrated on assessing the antioxidant efficacy of nano-hesperidin, illustrating its capability to mitigate oxidative stress [134]. Nonetheless, investigations into nano-hesperidin for anti-acne purposes remain few. Notwithstanding its anti-inflammatory and antibacterial attributes, additional research is required to investigate the efficacy of nano-hesperidin as a treatment for acne, presenting a novel approach for more effective and tailored acne therapies.

### 9.4. Nobiletin

Nobiletin, a flavonoid sourced from citrus fruits, demonstrates anti-inflammatory and antibacterial properties that may assist in acne treatment. Research has been conducted on nobiletin nanoparticles for enhancing skin penetration and their anticancer properties [135,136]. The utilisation of nobiletin-loaded nanoparticles for acne therapy remains little investigated. Investigating nobiletin nanoparticles may provide novel therapeutic avenues for acne sufferers by addressing the inflammation and microorganisms implicated in acne aetiology.

### 9.5. Epigallocatechin Gallate (EGCG)

Epigallocatechin gallate (EGCG), a principal ingredient in green tea, has been extensively researched for its antioxidant, anti-inflammatory, and antibacterial characteristics. Epigallocatechin-3-gallate (EGCG), a notable polyphenol in green tea, has attracted considerable interest for its potential as an anti-acne agent owing to its diverse biological properties. The development of *Acne vulgaris* is intricately linked to the proliferation of *Propionibacterium acnes*, inflammation, and oxidative stress [137].

Furthermore, employing nanotechnology for the delivery of EGCG may mitigate the adverse effects typically linked to conventional acne therapies, including skin irritation and dryness. This is especially pertinent in light of the increasing apprehension regarding antibiotic resistance and the detrimental consequences of systemic therapies such as isotretinoin [138].

Prior studies indicated that EGCG can diminish sebum production, regulate critical physiological pathways, and specifically target acne-inducing bacteria, such as *Propionibacterium acnes*. Furthermore, the findings of an 8-week clinical investigation indicated that EGCG was beneficial in alleviating acne symptoms and was well tolerated by participants. These data indicate the viability of EGCG as a natural alternative for acne treatment [6].

Although research on EGCG nanoparticles has been undertaken, the specific investigation of EGCG-loaded nanoparticles for acne treatment remains insufficiently addressed. The formulation of EGCG nanoparticles for acne may offer a more effective means of administering this potent ingredient to address acne lesions.

Regulatory challenges are present in the licencing of nanoparticle-based delivery systems in dermatological treatments, as these systems are a relatively new approach and may require complex formulations [139]. A few of the most significant regulatory obstacles are as follows:

- Due to the distinct behaviours of nanoparticles in the body relative to bulk materials, it is essential to conduct long-term safety studies to evaluate potential risks, including skin irritation, systemic toxicity, and tissue accumulation. Nanoparticles exhibit varying behaviours influenced by their size, surface charge, and material composition, necessitating comprehensive toxicological investigations [140].
- Regulatory agencies require comprehensive in vivo testing to evaluate the skin penetration and possible systemic absorption of nanoparticles. Additionally, cytotoxicity, sensitisation, and irritation tests are required [141].
- It can be difficult to achieve uniformity in the size, shape, and surface properties of nanoparticles, but maintaining consistency across batches is crucial for regulatory approval. Nanoparticle-based delivery systems are frequently sensitive to minute changes in production parameters, such as temperature, pH, and raw material quality [142].
- Strict rules must be followed in characterising nanoparticles (regarding the size, charge, form, stability, etc.). Although strong quality control policies and standardised testing techniques are demanded by regulatory authorities, these processes are still under development thus it is challenging to provide unambiguous regulatory routes [142].
- Regulators mandate comprehensive data regarding the capacity of nanoparticles to permeate the skin and their potential to access deeper layers, including the dermis or beyond. Assessing the ability of nanoparticles to penetrate the skin barrier and evaluating their potential for damage or systemic exposure presents a significant problem [62].

## 10. Conclusions

This study examined the utilisation of nanoparticles encapsulated with plant polyphenols and flavonoids as a more efficient delivery mechanism for anti-acne treatment. The findings indicated that nanoparticles enhance the bioavailability, stability, and efficiency of plant polyphenols and flavonoids, which possess antioxidant, anti-inflammatory, and antibacterial properties advantageous for acne treatment. Encapsulating these bioactive compounds in nanoparticles considerably enhances their efficacy in treating acne, as nanoparticles provide the controlled release of active compounds and improve penetration into deeper skin layers.

The nanoparticles improve the delivery of these phyto-compounds, as well as their stability and bioavailability, facilitating skin penetration and enabling the attainment of an in situ therapeutic concentration at the application site. Moreover, the multifunctional mechanisms of polyphenols and flavonoids encompass anti-inflammatory and antimicrobial properties, regulate sebum production, and enhance skin regeneration, highlighting their efficacy as anti-acne agents. Additionally, their untested safety profile renders them particularly appealing to patients with delicate skin or for long-term treatment options.

The advancements in polyphenol and flavonoid-loaded nanoparticles signify a new age in acne treatment. Ongoing investigation of these natural agents, coupled with innovative delivery methods, should provide optimism for enhanced patient outcomes and quality of life, fostering a more comprehensive strategy for the treatment of *Acne vulgaris*. Embracing these advancements will be crucial for redefining acne therapy and enhancing general skin health in the future.

## Figures and Tables

**Table 1:** Conventional products as anti-acne agents.

Compound	Intervention	Reference
Retinoids	Topical retinoids may be utilised as monotherapy for inflammatory acne, in conjunction with more severe acne types, or as a maintenance therapy. They typically regulate the development of microcomedones, diminish the growth of lesions and existing comedones, limit sebum secretion, and normalise epithelial desquamation. They focus on microcomedones and inhibit comedone development. They may also exhibit anti-inflammatory activities.	[30]
Salicylic Acid	As a keratolytic drug, salicylic acid breaks down the intercellular cement that holds the cells of the epithelium together under the skin. It reduces inflammation a little, helps some substances work better, and in small amounts, it stops fungus and bacteria from growing. A lot of over-the-counter acne medicines contain salicylic acid.	[31]
Benzoyl Peroxide	Benzoyl peroxide is a topical antiseptic, initially utilised as a peeling agent for acne treatment. It exhibits many qualities, functioning as both a comedolytic and an antibacterial agent, without influencing sebum production. Benzoyl peroxide exhibits bactericidal efficacy against *P. acnes* by liberating free-radical oxygen, which destroys bacterial proteins.	[32]
Azelaic Acid	Azelaic acid is a naturally occurring dicarboxylic acid that obstructs protein synthesis in the *P. acnes* species. It is an efficacious agent due to its bacteriostatic, anti-inflammatory, antioxidant, and anti-keratinizing effects.	[33]
Sulphur	Sulphur has been frequently employed in acne preparations in the past. Nevertheless, this active substance has become unpopular as a result of its unpleasant odour. Sulphur is a chemical that has been shown to possess moderate bacteriostatic and keratolytic properties. Sulphur is reduced to hydrogen sulphide within the keratinocytes, which is purported to be responsible for the degradation of keratin in the epidermis.	[34]
Topical Corticosteroids	Topical corticosteroids may be utilised in specific cases, such as the treatment of severe inflammatory acne. The treatment duration should be brief, and evidence of their efficacy must yet be established.	[5]

**Table 2:** Mechanisms of flavonoid and polyphenol compounds in the antibacterial and anti-inflammatory treatment of acne.

Compound	Structure (Chemdraw)	Mechanism	Reference
Cathecin	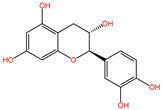	Catechins, especially epigallocatechin gallate (EGCG), show significant antibacterial activity against acne-causing bacteria such as *Staphylococcus epidermidis*, *Staphylococcus aureus*, and *Propionibacterium acnes*. Catechins can also inhibit enzymes involved in the pathogenicity process, such as protein tyrosine phosphatase and cysteine proteinases. Catechins have anti-inflammatory properties which can help reduce the inflammation that often occurs in acne-prone skin. Catechin can affect the metabolism of the sebaceous glands, which play a role in sebum production.	[49]
Curcumin	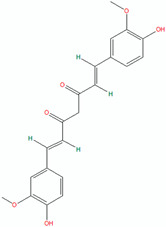	Curcumin protects against skin damage caused by prolonged UVB exposure accelerates wound healing and supports collagen deposition. It also increases the density of fibroblasts and blood vessels in injured areas.	[50]
Coumarin	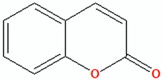	Coumarin, extracted from *Sanguisorba officinalis* L. roots, exhibited strong anti-acne qualities.	[51]
Lichocalcone	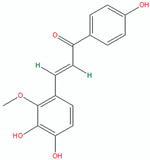	Licochalcone A inhibited *P. acnes*-induced activation of the NLRP3 inflammasome in macrophages and sebocytes, leading to reduced production of caspase-1 and IL-1β. Topical application of licochalcone A attenuated *P. acnes*-induced skin inflammation in a mouse model, as evidenced by reduced inflammatory cell infiltration, swelling, and ear thickness. The anti-inflammatory effects of licochalcone A were mediated by suppression of the NLRP3 inflammasome, as shown by reduced caspase-1 activity and IL-1β production in the mouse ear tissues.	[52]
Orobol	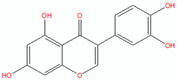	Orobol, a metabolite of genistein, inhibited the *P. acnes*-induced increases in IL-6 and IL-1α levels in human keratinocytes more effectively compared to salicylic acid. Orobol decreased the IL-1α and IL-6 mRNA levels and inhibited the phosphorylation of IKK, IκBα, and MAPK induced by *P. acnes*. Orobol decreased the expression of Ki67, indicating it reduced the hyperproliferation of keratinocytes induced by *P. acnes*.	[53]
Phloretin	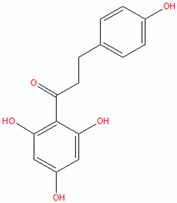	Phloretin has antimicrobial activity against *P. acnes*, *P. granulosum*, and *S. epidermidis*. Phloretin attenuates *P. acnes*-induced inflammation by inhibiting COX-2 and PGE2 expression. In a clinical trial, phloretin treatment led to a significant reduction in acne lesions, sebum output, and porphyrin levels.	[54]
Resveratrol	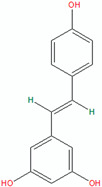	Resveratrol demonstrated sustained antibacterial activity against *P. acnes*. Resveratrol was significantly less cytotoxic to monocytes and keratinocytes compared to benzoyl peroxide.	[55]

**Table 3:** Efficacy of flavonoid and polyphenol nanoparticle formulations in acne treatment.

Compound	Intervention	Types of Nanocarrier	Conclusion	Reference
Curcumin	The intervention was the preparation of curcumin nanoparticles (nanocurcumin) using a wet-milling technique. Curcumin (100 mg) was dissolved in dichloromethane, sprayed into boiling water under ultrasonic conditions, and then, the solution was concentrated and freeze-dried to obtain the nanocurcumin powder.	Polymer-based nanocarriers	Nanocurcumin showed improved antimicrobial activity compared to regular curcumin, with lower minimum inhibitory concentrations against various bacterial and fungal strains.	[114]
Resveratrol	Nanoemulsion gel containing adapalene and 5 mg of resveratrol (F1), applied once daily. Nanoemulsion gel containing adapalene and 10 mg of resveratrol (F2), applied once daily.	Surfactant-based nanocarrier	The prepared nanoemulsion gel formulation containing adapalene and resveratrol showed better efficacy in treating acne pustules compared to the marketed formulation. The treatment time was significantly shorter for the prepared formulations.	[115]
Quercetin	Various nanoformulations were developed to deliver quercetin, including polymer-based, lipid-based, surfactant-based, cyclodextrin-based, and inorganic nanoparticles.	Lipid-based nanocarriers	Lipid-based nanocarriers like liposomes and nanostructured lipid carriers improve quercetin’s oral absorption, targeting, and antioxidant activity.	[110]
Mangosteen	0.5% mangosteen nanoparticle loaded gel (MNLG) or 1% clindamycin gel, applied twice daily to one side of the face.	Lipid- and polymer-based nanocarriers	MNLG significantly reduced both comedone and inflammatory acne lesions by 66.86% and 67.05%, respectively, after 12 weeks of treatment, which was comparable to the 55.33% and 64.16% reduction seen with 1% clindamycin gel. MNLG showed significantly better improvement in clinical severity compared to 1% clindamycin gel at week 12. Both MNLG and clindamycin gel significantly reduced porphyrin levels, which indicates a reduction in *P. acnes*, with no significant difference between the two groups.	[116]
	Mangostin nanoparticle gel (1.2% *w*/*w* a-mangostin) applied twice daily (morning and night, after shower) for 28 days, with approximately 3 mL of gel applied to each half of the face (0.025 mL/cm^2^ coverage).	Lipid-based nanocarriers	The mangostin nanoparticle gel showed significantly greater improvements in both acne severity index (ASI) and inflammatory lesion count compared to the placebo gel base,	[117]
Flavonoid	Minimal inhibitory concentration of AgNPs for acne-causing agent *P. acnes* at 3.1 μg/mL		Efficacy of green synthesised silver nanoparticles using flavonoid-containing *Coriandrum sativum* leaf extract as antacne	[118]
